# An Approach to Quantify the Float Effect of Float Serves in Indoor and Beach Volleyball

**DOI:** 10.3389/fspor.2020.559277

**Published:** 2020-10-06

**Authors:** Mathias Reiser, Karen Zentgraf, Stefan Kindermann, Stefan Künzell

**Affiliations:** ^1^Neuromotor Behavior Laboratory, Department of Psychology and Sport Science, Justus-Liebig-University Giessen, Giessen, Germany; ^2^Department of Movement Science and Training in Sports, Institute of Sports Sciences, Goethe-University Frankfurt, Frankfurt, Germany; ^3^Department of Population Health Sciences, German Center for Neurodegenerative Diseases, Bonn, Germany; ^4^Institute of Sports Sciences, University of Augsburg, Augsburg, Germany

**Keywords:** kinematics, motion analysis, biomechanics, sport, aerodynamics

## Abstract

The float serve is an effective weapon to impede the attack of the opposing team. Because of its great importance in indoor and beach volleyball, we measured and quantified the float effect. We recorded 24 float serves of 12 top athletes in beach volleyball and indoor volleyball, respectively, and analyzed them using video analysis. We determined the 3D trajectories of the ball flight and developed two measures to describe the size of the float effect, the mean residuals and the anticipation error. Both were derived from regression models. These measures suggest that the float effect is greater in the vertical plane than in the horizontal plane, both for indoor and beach volleyball. Analyses of ball release velocities suggest that a certain ball release velocity is a necessary, but not sufficient, condition for ball floating. A validation of the float measurements with subjective expert ratings showed a correlation with the horizontal deviations. This study provides a new approach to analyze floating in on-court volleyball serves and broadens the knowledge for float effects in sports.

## Introduction

In volleyball, the serve—as the beginning of a rally—aims at making the preparation of the opposite team's attack as difficult as possible. A poor receive in the opposite team increases the chances to score a point, either by scoring an ace or by reducing the effectiveness of the opponent's attack. Therefore, in the history of volleyball, coaches, and players have tried to develop serving skills that focus on either increasing ball speed (e.g., the jump serve) or on producing unpredictable ball trajectories, e.g., the float serve and the jump float serve (henceforth: float serve) to make ball reception more difficult (MacKenzie et al., 2012). With regard to the second aspect, the float serve has become more popular during the last two decades. According to several studies, the usage of the float serve at the elite level has increased in both beach and indoor volleyball (Künkler, 2004; Moras et al., 2008; Zimmermann and Thorsteinsson, 2008; Koch and Tilp, 2009; Zimmermann, 2009). In the 2008 Olympics, around 30% of all serves in the men's competitions have been classified as float serves, compared to only 15% in the 2004 Olympics. At the beach volleyball World Championship in Stavanger (Norway) 2009, around 40% of all serves were float serves (Künkler, 2009). The percentage seems to be even higher in women's competitions. At the beach volleyball Grand Slam in Klagenfurt (Austria) in 2008, around 80% of all serves were float serves, 62.5% of those without a jump, and 38.5% with a jump (Koch and Tilp, 2009). A recent study by López-Martínez et al. (2020), which analyzes more than 3,000 serves of elite-level women beach volleyball players, confirms this observation. The jump float serve (44.8%) and the standing float serve (32.8%) showed a higher occurrence than the jump power serve (22.3%). The advantage of the float serve is that, although the reception difficulty is high, the error rate is lower compared to the jump serve (López-Martínez et al., 2020).

In the jump serve, aiming to achieve high ball speed, players hit the ball at the peak of their jump with a dynamic arm action that induces forward rotation of the ball (topspin) (Huang and Hu, 2007; MacKenzie et al., 2012). In contrast, a float serve requires a short contact between the stiff hand and the ball at its center without imparting rotation to induce sudden directional changes in the horizontal and vertical planes (Huang and Hu, 2007; MacKenzie et al., 2012; Czimek, 2017). The rationale is that predicting the ball trajectory, necessary to prepare a reception that delivers the ball accurately to the setter (Paulo et al., 2016), should then be made more difficult and error prone. Overall, the effectiveness of float serves in volleyball should be related to distance between the expected and the actual position of the ball during the flight time.

(Goff, 2013) carefully reviewed the aerodynamics of the sports projectiles and concluded that unstable airflow is the cause of the sudden change in ball trajectory other than the Magnus effect. One prominent hypothesis is that due to abrupt transitions from laminar to turbulent airflow, air resistance changes causing a drag crisis (Cairns and Van Lierop, 2000; Cairns et al., 2000; Cairns, 2004). This, however, depends upon physical interactions of ball speed, of (lacking) ball rotation, and other factors (e.g., ball type and orientation of the surface pane). Currently, a model considering all these aspects is missing. Therefore, the prediction of the unstable airflow conditions causing the ball to float is rather unreliable, even when the initial conditions are well known (Wei et al., 1988). The exact mechanical description of floating balls is complex and has been a matter of debate in physics and mathematics for many decades (Mehta, 1985; Fuchs, 1991; Kao et al., 1994).

**Figure 1:**
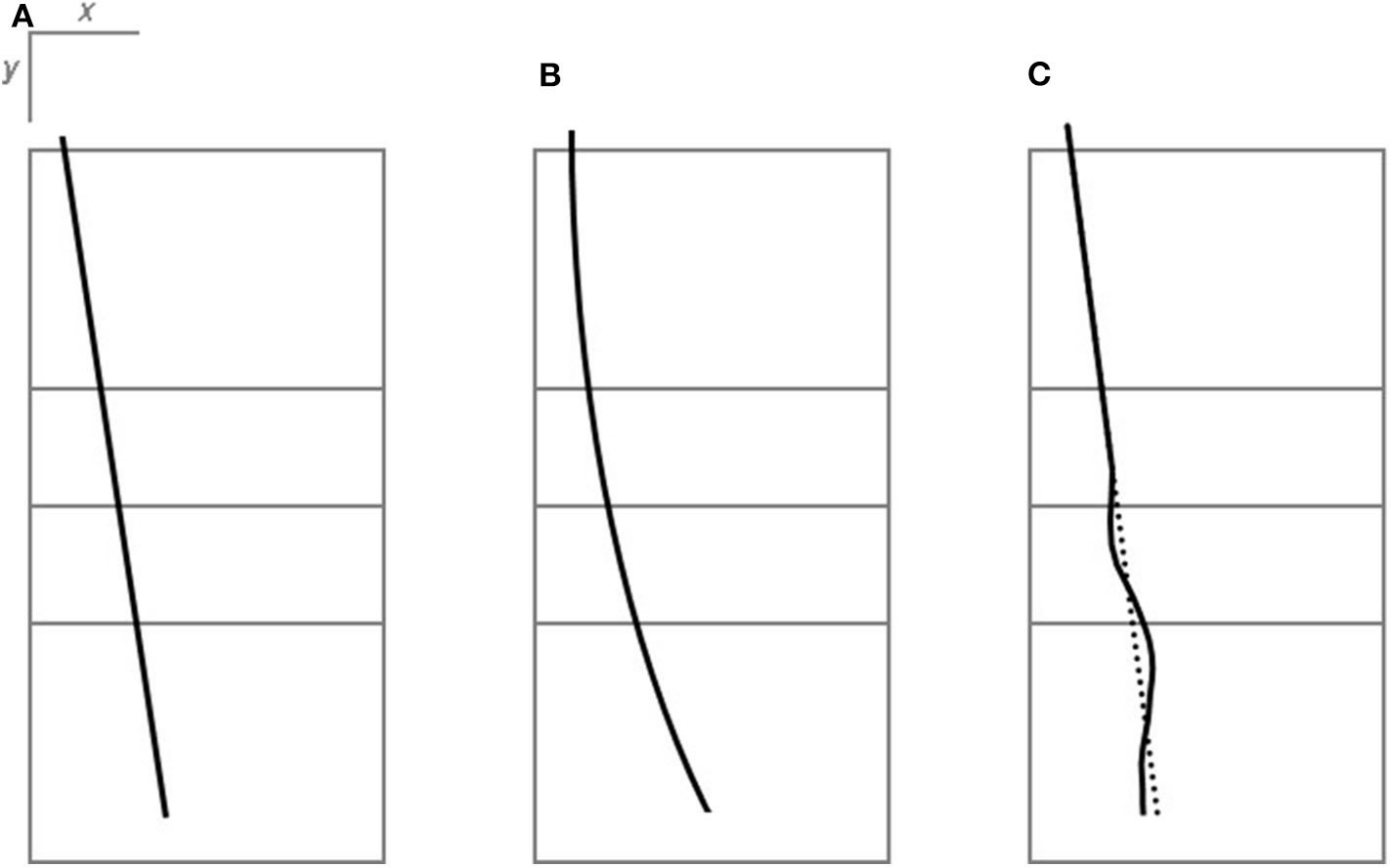
Prototypical ball trajectories of served balls. All figures are presented from a bird's eye view to depict changes in the lateral direction. **(A)** Straight serve: The horizontal acceleration is zero. The ball moves with constant velocity in the horizontal plane. **(B)** Serve with side spin: The horizontal acceleration is constant yet not zero. The ball travels with increasing velocity in the horizontal plane. **(C)** Float serve: The horizontal acceleration changes unexpectedly at a certain location. The ball, initially traveling with a constant velocity in the horizontal plane, experiences sudden accelerations in the horizontal plane, and deviates from the originally straight path (dotted line).

To the best of our knowledge, so far, no procedure is known to quantitatively describe the extent of random movement in the ball flight path resulting from a successful float serve. However, subjective descriptions of floating ball paths from experienced coaches and players suggest some requirements for a float effect measure. In Figure 1, three prototypical ball trajectories resulting from a volleyball serve are presented from an aerial viewpoint. Concerning the straight serve (see Figure 1A, typical ball path of a serve with top-spin), horizontal acceleration perpendicular to the ball flight direction remains zero during ball flight. A curved trajectory (Figure 1B) results, when the acceleration perpendicular to the flight direction is constant but does not equal zero (typical course of a serve with side-spin). The initially straight ball trajectory of a float serve (Figure 1C) may deviate unpredictably to the left or to the right (sudden deviation in the horizontal direction). In this case, the horizontal acceleration changes in a jerky manner at a certain point during the ball flight. A float serve can also lead to an unpredictable path in the vertical direction, perceived as a sudden “falling” of the ball.

Using an impact-type ball-ejection device, Asai et al. (2010) assessed float effects of ball trajectories with ball-flight velocities near the Reynolds number and calculated the deviation of the landing points' 2D coordinates of repeated trials as a measure of aerodynamic instability. However, kinematic data of on-court ball trajectory served by athletes is still lacking. Therefore, the purpose of the present study is to implement a measurement procedure for quantifying the extent of float of volleyball serves. The main objective is to establish kinematic-based measures by recording and analyzing ball flight curves generated by experienced volleyball players close to competition conditions. The method was applied in beach volleyball as well as in indoor volleyball, as the float serve is a relevant technique in both sports. In addition, we test the hypotheses that (1) ball release velocity is a predictor of the extent of floating and (2) that objectively, kinematic-based measures correlate with expert's subjective ratings of the perceived extent of floating of the ball.

## Methods

### Study Design and Participants

To quantify the extent of random movement (float effect) in the ball flight trajectories of serves by experienced volleyball players, two studies (beach volleyball, study 1; indoor volleyball, study 2) were carried out. Five young elite beach volleyball players (3 males) and one senior volleyball player (male) participated in study 1. According to a taxonomy suggested by Swann et al. (2015), three of the young players were competitive elite, two were successful elite, and the senior was a world-class elite player. Six successful elite indoor volleyball players (all females) participated in study 2. From each participant, the ball flight paths of 24 float serves were analyzed using video.

Each participant performed two series of 12 float serves using his or her individually preferred float serve technique (float serve or jump float serve). For each float serve, the parameters of the ball flight (ball velocity, release angle, and release height) were determined and a measure was calculated that quantifies the unpredictable portion of the movement trajectory. In addition, for each floating serve, the subjectively perceived floating of the ball was documented from the perspective of the reception player and the coach.

The study was not an experimental intervention. Instead, data were collected during a usual training session on request of the national coach. The serves were performed at the beginning of the training following a standardized warm-up routine. The participants are voluntary members of the national team and, as such, participate voluntarily in the training session. With regard to the task assigned to the participants (2 × 12 float serves), we did not see any special requirements from the study that goes beyond the normal training load and that would make a vote by an ethics committee necessary. In principle, in research projects with German sports associations (here the German Volleyball Association), the ethical adequacy for the welfare of the athletes is ensured bilaterally and jointly.

### Data Acquisition

**Figure 2:**
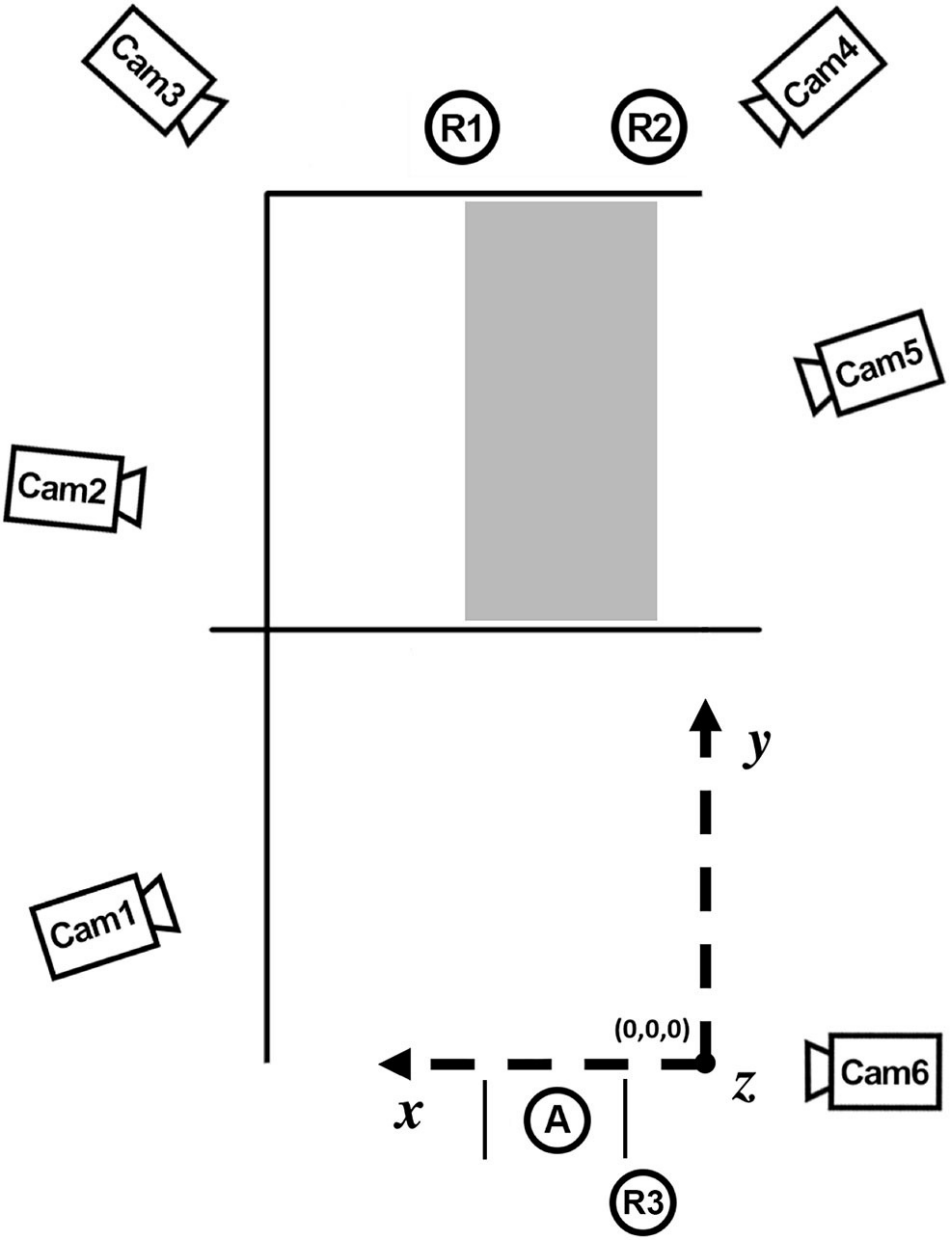
Camera setup and coordinate system from a bird's eye view. The volleyball court, camera positions (“cam”), the position of the server (A), the serving area, the target corridor (gray), and the position of the raters (R1, R2, R3) are shown. The origin of the coordinate system is located in the right corner of the base line of the serving player's court, seen from the server's perspective.

Study 1 was carried out on an indoor beach volleyball court at the Olympic training center, Stuttgart, Germany; study 2 took place at the Olympic training center, Berlin, Germany. Set up of the cameras was similar in both studies (see Figure 2). The court dimensions were in accordance with the official volleyball rules (INDOOR: 9 × 18 m; BEACH: 8 × 16 m). The net heights were 2.24 m for the INDOOR court and 2.35 m for the BEACH court. Officially approved balls were used (INDOOR: MIKASA MVA 200; BEACH: MIKASA Beach-Volleyball-Beach-Champ VLS 200 Micro).

For the acquisition of kinematic data, five video cameras (Basler Aviator) were used to record the trajectory of the ball flight. With the sampling rate of 100 Hz, the cameras allow a spatial resolution of 1,600 × 400 pixels, which was adequate for reliably digitizing the center of the ball. An additional video camera (camera 6) was placed in a way to record the sagittal motion of the float serve movement. Both indoor courts offered sufficient light conditions allowing short shutter speeds (≤1/250 s). For 3D reconstruction of the ball trajectory, a reference system with 32 pass points was used. Reference system was introduced by using a 5-m telescopically extendable leveling rod made from aluminum profiles (NESTLE GmbH, Dornstetten, Germany) that was mounted vertically on a tripod on which clearly visible reference points were marked at fixed distances. At a total of eight locations within the capture volume, the leveling rod was aligned vertically, and then the positions of the reference points relative to the origin were measured using a laser rangefinder (GLM40, Bosch, Germany). Vertical alignment was achieved by means of spirit levels and optical bearing. For this purpose, the leveling rod was carefully adjusted according to the guidance of two observers from two different viewing angles (perpendicular to each other), who visually controlled for exact alignment of the rod with reference to clearly visible vertical house edges. From each measuring position of the leveling rod, a video frame was captured from each camera perspective. The calibration images needed for each camera were then generated by video overlay (software Gimp 2.6) of the respective video frames.

### Task and Procedure

The athletes got together in pairs to participate in the investigation. While one of the two players performed 12 float serves, the other player acted as a rater (R1). Together with the volleyball coach (Figure 2, R2), the rater was positioned behind the opposite service line (see Figure 2) and rated each float serve of his or her partner. A second coach (R3) was positioned inclined behind the float server and also was asked to evaluate the serves in terms of the magnitude of floating. All participants independently rated the magnitude of the ball floating on a 5-point scale from 0 (“no floating at all”) to 4 (“very strong floating”). They were instructed to evaluate the “overall impression” of a perceived floating of the ball, independent of the floating direction. When serves were going off-bounce, they were not rated.

The server was instructed to serve within a 3-m-wide area and to hit a target corridor in the rear section of the opposing field (i.e., in x-coordinates between 1.5 and 5.5 m) (see Figure 2). The target corridor was illustrated to the players on a tactical board, but there were no reference lines on the court. Whether or not the players served the ball into the target area was determined afterward based on the 3D coordinates of the ball flight curve. All serves that hit the target corridor were included in the analysis.

When approval was given, the video recordings started, and the serve could be executed. The synchronized video recordings of the ball flight were triggered by the record stop of the takes, which was handled manually when the ball touched the ground. The last 4 s of the video data prior to the record were stored. Data storage of the six video files at a time took ~40 s. During this time, the three raters documented their perceived floating of the ball curve. The required time period for data acquisition of a series of 12 float serve per server was ~10 min.

### Acquisition of the Ball Flight Trajectories

For the acquisition of the kinematic data of the ball, the 2D coordinates of the center of the ball were manually tracked in the videos of all cameras at each frame. To minimize tracking inaccuracies, a circular mask was superposed to the video display, using the center of the circular mask as a reference for the center of the ball. These mask completely enframed the ball and, thereby, allowed precise determination of the 2D positions of the ball center. Tracking started five frames prior to ball-hand-contact of the serve and was finished five frames after ball-ground contact.

**Figure 3:**
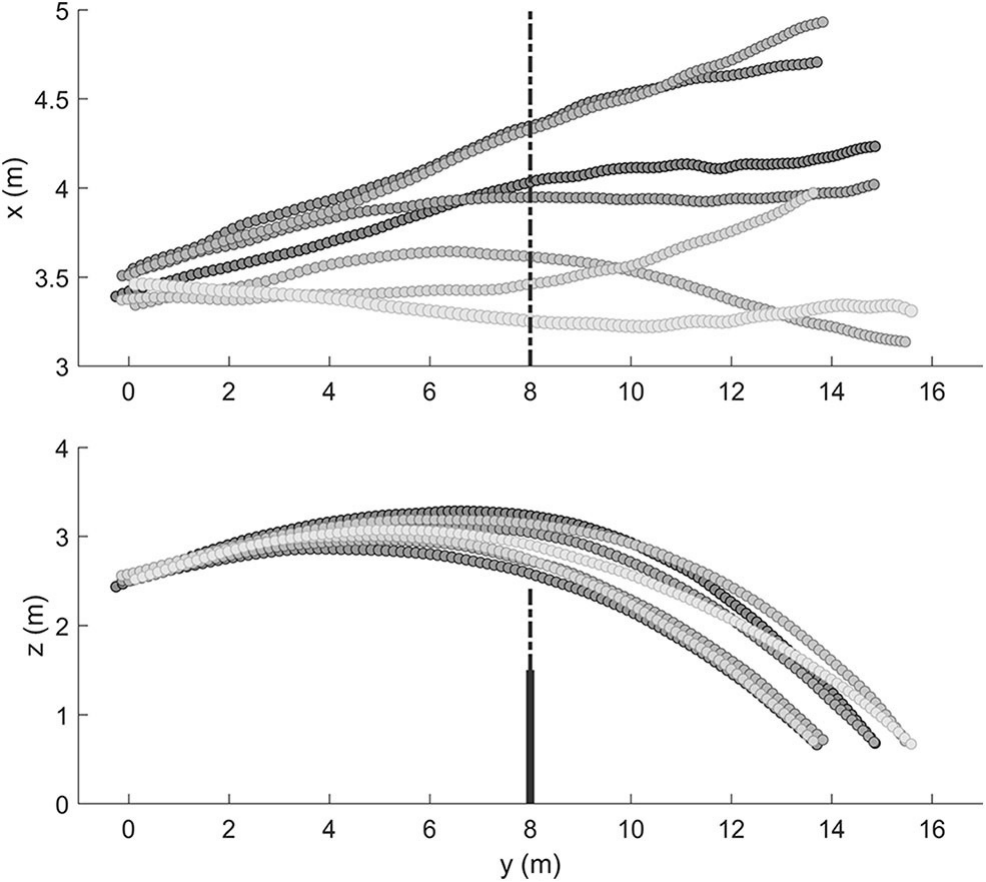
Representative trajectories of a series of float serves in beach volleyball. Shown are the 3D coordinates of the ball flights from a bird's eye view (above) and from the side view (below).

Computing of the 3D-coordinates of the ball flight was performed using the Peak Motus Software (Version 9, Vicon Motion Systems. Inc.). All further data analyses were performed using MATLAB^©^. First, the raw data were read out from Motus files. Thereafter, the beginning and end of the to-be-analyzed section of the ball flight was determined. The start frame of this selected section was defined as the frame directly after ball-hand contact. Data sets end when the vertical distance between the ball flight trajectory and the ground was smaller than 0.7 m. Both events were detected automatically using the velocity data (start) and the position data (end) of the ball. After the cutting procedure, the 3D coordinates were low-pass filtered with a cutoff frequency of 10 Hz using a zero-lag digital filter, minimizing start-up and ending transients. The goodness of the filter was based on visual inspection of the results (velocity profiles of the ball trajectories). Therefore, the filtered trajectories were plotted against the raw data. With the chosen cutoff frequency, measurement noise was reduced significantly, while the characteristics of the ball flight parameters of interest were still visible. Since we do not expect more than 10 visually perceptible changes of direction with the given mass of a volleyball and flight durations of just under 1 s, we assume that all relevant regular (predictable) as well as all unexpected (floating) movement components will pass the filter and be preserved at the selected cutoff frequency. In Figure 3, the 3D coordinates of a series of float serves are plotted.

To determine the release height (h_0_), release angle (α_0_), and velocity (v_0_) of the ball, a nonlinear regression was applied using the function for projectile motion

(1) $$\begin{array}{r} z=\frac{-g}{2v_{0}^{2}\cos^{2}\alpha_{0}}y_{dir}^{2}+(\text{tan}\alpha_{0})y_{dir}+h_{0} \end{array}$$

The fit was done for the 3D ball coordinates projected to the ball flight direction, which is given by the direction from the start to the end position of the ball flight, where *g* represents gravity and *y*_*dir*_ the distance covered by the ball in the flight direction.

### Calculation of the Dependent Measures

The assumption behind our calculations is that the ball flight trajectory is composed of a regular, predictable proportion and a floating, unpredictable proportion. The predictable proportion can be captured by a polynomial regression, while the floating proportion is estimated by the deviation from the regression curve. For the vertical plane along the side-line (the plane formed by the Y–Z axes), it can be assumed that a ball flight trajectory without any random movement follows a parabolic curve (ignoring the air resistance). The horizontal plane formed by the X–Y axes described the medial–lateral deviation of the ball trajectory (see Figure 3). In this case, if the ball receives a small lateral spin, this would cause an acceleration in the horizontal direction resulting in a curved ball trajectory. Thus, for the horizontal plane, we do not compute linear regressions because a continuously curved trajectory is not classified (due to its predictability) as a floating ball. The curvature of such a ball trajectory in the horizontal plane is also best described by a quadratic regression.

**Figure 4:**
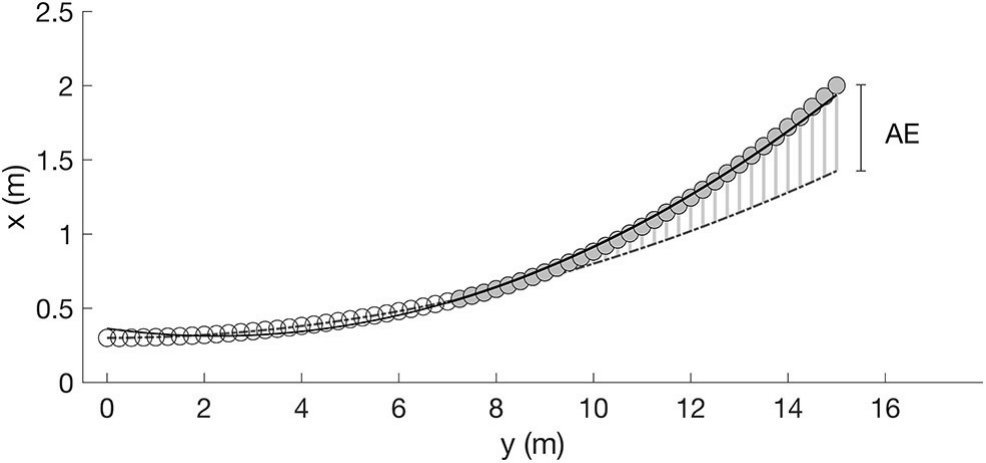
Illustration of the floating measures using a hypothetical data set. Flight path without ball floating (open circles) and after the ball receives an additional acceleration (gray circles). Mean residuals: Ball floating is indicated by a worse fit of the position data of the entire trajectory (solid line). Mean deviation from the regression line (x.R) is used to quantify the extent of ball floating. Anticipation error (AE): Based on the polynomial regression of the first 7 m of the ball trajectory, the flight path is extrapolated (dash-dotted line) to the (“anticipated”) locus of reception and the horizontal and vertical distance to the actually measured locus of reception is computed.

We computed two measures of the deviation, the mean residuals (x.R) and the spatial distance at the locus of reception (“anticipation error”). In Figure 4, both measures are illustrated using a hypothetical data set. It shows a curved ball trajectory (*x* = 0.005^*^y^2^ + 0.3) from a bird's eye view (horizontal plane) without any random movement over the first 7 m (open circles). Furthermore, we assume that there was no measurement error when capturing the 3D coordinates of the ball flight.

#### Mean Residuals

For the first section of the ball path (0–7 m), the quadratic regression perfectly fits the position data (dash-dotted line in Figure 4), so that the residuals (e.g., the deviations of the observed coordinates from the coordinates predicted by the statistical model) are all equal to zero (x.R = 0 cm). If the ball receives an additional random float acceleration during ball flight (in the example after 7 m), the flight path deviates from the predetermined path in the further course (gray circles). Such a sudden change in direction is indicated by a worse fit of the entire trajectory (position data from y = 0 to y = 14.8 m) of the ball flight (solid line). In our example, the mean deviation from the regression line (solid) is x.R = 2.6 cm (see Figure 4). The mean residuals were calculated for the vertical (mR_vertical) and horizontal plane (mR_horizontal) separately. Additionally, for a “combined” mean residual measure, mR_vertical and mR_horizontal were summed up.

#### Anticipation Error

The second measure to describe the random proportion of the ball-flight path maps the situation of the reception player. We assume that the player observes the approaching ball for a certain path length and then predicts the (anticipated) locus of reception. We define the locus of reception as the position in space where the ball path enters a plane 70 cm above and parallel to the floor. Based on the polynomial regression of the first 7 m of the ball trajectory, we extrapolate the flight path to the defined (“anticipated”) locus of reception (dash-dotted line). The horizontal and vertical distance between the point of intersection actually measured and the anticipated locus of reception is the anticipation error. The “combined” anticipation error results from the Euclidean distance between the point of intersection actually measured and the anticipated locus of reception.

### Statistics

The interrater reliability of the experts rating the perceived ball floating was examined using the intraclass correlation coefficients (ICC, model: two-way random-effects, type: mean of *k* raters, definition: consistency). ICC estimates and their 95% confident intervals were calculated (Koo and Li, 2016). The Spearman rank order correlation coefficients were calculated to determine the relationship between the expert rating and the kinematic ball floating measures (mean residuals, anticipation error). A nonparametric statistical measure was used because the ratings are data on an ordinal scale. In order to analyze whether release velocity differs between individual athletes, one-way ANOVAs were calculated separately for INDOOR and BEACH. Bivariate regression analysis was used to establish the strength of the relationship between release velocity and both dependent variables. The statistical significance level was set to *p* < 0.05. Statistical calculations were run with SPSS 23.

## Results

In study 1 (beach), 94 serves (out of 144, six players with 24 serves each) could be analyzed. In study 2 (indoor), 92 serves (out of 144, six players with 24 serves each). The remaining serves were either net balls or “out”.

### Ratings

In both studies, the magnitude of perceived floating was mostly consistent between the three raters (beach: *ICC* = *0.71 (CI: 0.60–0.80), n* = 94; indoor: *ICC* = *0.79 (CI: 0.70–0.86), n* = 92). ICC estimates indicate moderate to good reliability. Since average judgments are generally more reliable and valid than individual judgments, sufficient agreement is assumed (Bortz and Döring, 2002). The rating data were pooled, i.e., the average of the three ratings was calculated.

Somewhat surprisingly, only few serves were judged as “strongly floating.” In study 1 (beach), just 18 serves (19.1%) were scored in this category; in study 2 (indoor), even less, only 8 out of 92 serves (8.7%). A comparison of the rating results for individual players (see Table 1) indicates that the ability to produce a floating ball path differs widely across players. The highest rating with mean = 2.3 points was found in the beach study (participant 1) and the lowest rating with mean = 0.5 points in the indoor study (participant 12).

**Table 1:** Anthropometric data, mean ratings, and release parameters (means, SD).

**Player**	**Age (years)**	**Height (m)**	**Serve type**	**Serve (*n*)**	**Rating (score)**	**Rel. velocity (m/s)**	**Rel. angle (degrees)**	**Rel. height (m)**
**Beach volleyball**								
1	18–20	1.78	JF	19	2.3	15.47 ± 0.66	10.56 ± 1.78	2.55 ± 0.03
2	18–20	1.81	JF	14	1.9	15.53 ± 0.87	10.54 ± 2.32	2.51 ± 0.03
3	21–25	1.87	JF	18	1.4	14.71 ± 0.57	11.47 ± 1.93	2.72 ± 0.03
4	46–50	1.90	SF	15	1.5	15.06 ± 0.58	13.15 ± 2.20	2.33 ± 0.04
5	21–25	2.02	JF	19	1.5	14.44 ± 0.64	12.17 ± 2.17	2.54 ± 0.02
6	21–25	1.92	JF	9	2.1	16.36 ± 0.53	8.00 ± 2.03	2.56 ± 0.17
Mean				94	1.7	15.15 ± 0.85	11.22 ± 2.45	2.54 ± 0.12
**Indoor volleyball**								
7	18–20	1.80	JF	15	1.6	17.77 ± 0.74	9.54 ± 2.12	2.44 ± 0.03
8	18–20	1.92	JF	22	0.9	16.79 ± 0.62	10.83 ± 2.49	2.56 ± 0.03
9	18–20	1.85	JF	14	2.0	19.06 ± 0.54	9.50 ± 1.89	2.58 ± 0.03
10	18–20	1.83	JF	12	1.0	18.62 ± 0.78	8.15 ± 1.91	2.65 ± 0.03
11	18–20	1.78	JF	18	1.6	17.73 ± 0.47	10.24 ± 1.61	2.57 ± 0.02
12	18–20	1.80	JF	11	0.5	17.39 ± 0.72	10.12 ± 2.70	2.56 ± 0.03
Mean				92	1.3	17.79 ± 0.99	9.87 ± 2.25	2.56 ± 0.07

*JF, jump float serve; SF, stand float serve*.

### Mean Residuals

**Figure 5:**
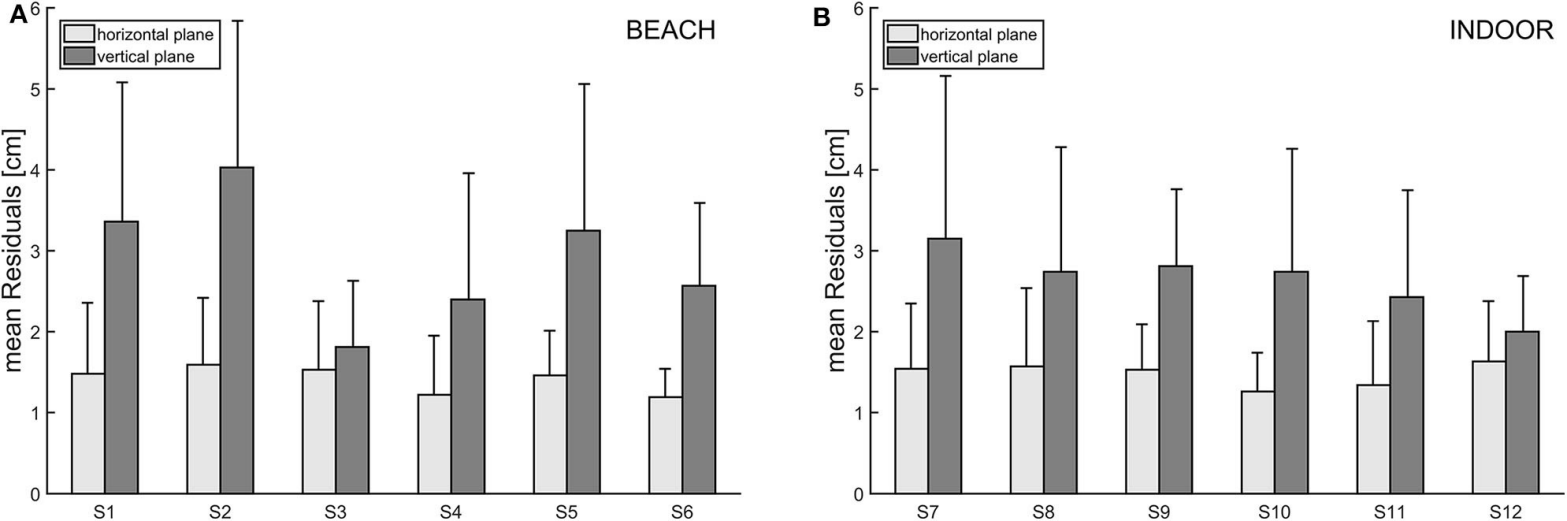
**(A,B)** Mean residuals for each participant of the beach volleyball (left) and indoor volleyball study (right). Bars are grouped due to deviations in the horizontal (light gray) and vertical (dark gray) planes.

The mean residuals represent the deviation between the observed ball path and the ball path estimated by a quadratic regression on the horizontal and the vertical plane, respectively. Overall (see Figures 5A,B), the mean residuals in the vertical plane (mR_vertical) are higher than in the horizontal plane (mR_horizontal). For indoor volleyball, mR_vertical (mean = 3.04 cm) is more than twice as high as mR_horizontal (mean = 1.44 cm), and for beach volleyball mR_vertical (mean = 2.70 cm) is approximately twice as high as mR_horizontal (mean = 1.46 cm).

### Anticipation Error

**Figure 6:**
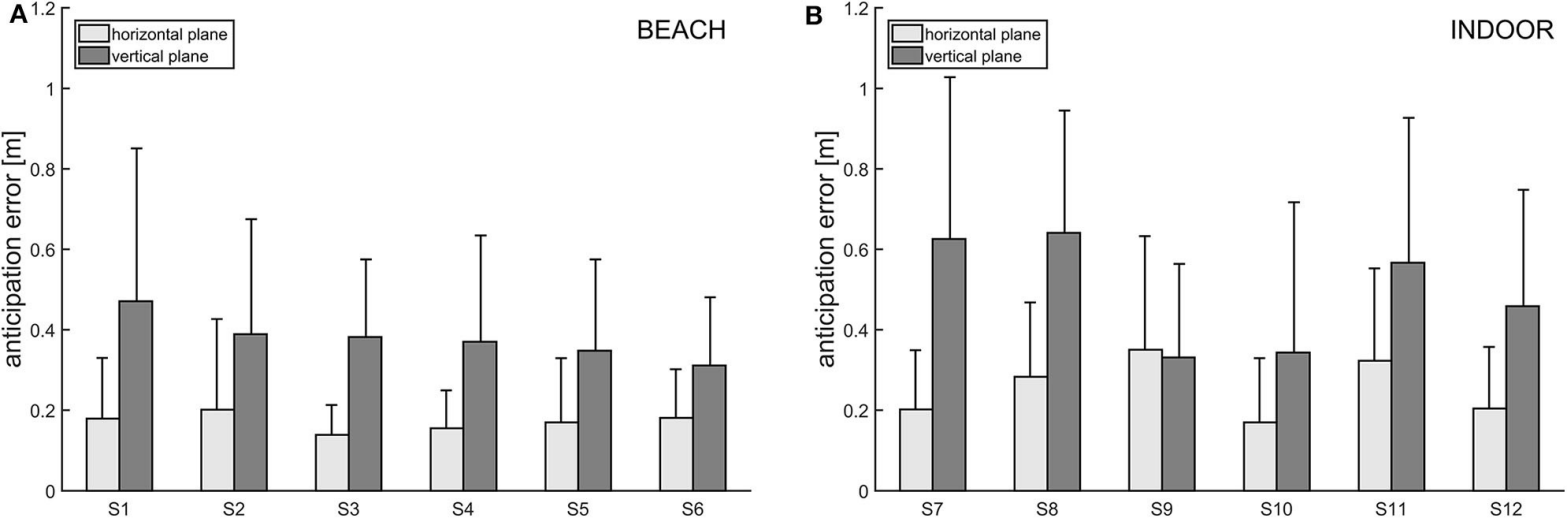
**(A,B)** Anticipation error for each participant of the beach volleyball (left) and indoor volleyball study (right). Bars are grouped for horizontal (light gray) and vertical (dark gray) deviations.

Overall, and similar to the mean residual measure, the anticipation error, too, indicates higher floating for the vertical plane compared to the horizontal plane (see Figures 6A,B). On average, for the vertical plane, the actually measured locus of reception deviates 0.39 m (SD = 0.27 m, beach) from, respectively, 0.52 m (SD = 0. 35 m, indoor) from the “anticipated” locus of reception. The largest measured anticipation error of a single float serve in the vertical plane was 1.50 m (INDOOR, participant 7). In the horizontal plane, anticipation error was 0.26 m (SD = 0.21 m, INDOOR), respectively, 0.17 m (SD = 0.14 m, beach), on average. In that plane, the maximum measured anticipation error was 0.99 m (INDOOR, participant 11).

### Correlations Between the Objective Floating Measures and the Subjective Ratings

In the first analysis, the ratings were correlated with the “combined” float measures. In the BEACH study, the mean residuals correlate significantly with the expert rating (*r*_*s*_ = 0.238, *p* = 0.021, *n* = 94), whereas no correlations were found for the anticipation error.

In the second analysis, the ratings were correlated with the vertical and horizontal components of the respective dependent measures. Rank correlations of expert rating with mean residuals in the horizontal plane show a moderate relationship for both studies (Spearman-Rho; BEACH: *r*_*s*_ = 0.277, *p* = 0.007, *n* = 94; INDOOR: *r*_*s* =_0.335, *p* = 0.001, *n* = 92), whereas for the vertical plane, no significant correlations were found (BEACH: *r*_*s* =_0.115, *p* = 0.269, *n* = 94; INDOOR: *r*_*s*_ = −0.077, *p* = 0.465, *n* = 92). For the anticipation error, a moderate but significant correlation with the expert rating was found for the horizontal plane. Rank correlation of anticipation error with expert rating was *rs*_=_0.232 (*p* = 0.024, *n* = 94; BEACH), and *r*_*s*_ = 0.301 (*p* = 0.003, *n* = 92; INDOOR). For the vertical plane, no significant correlations were found (BEACH: *r*_*s*_ = 0.050, *p* = 0.630, *n* = 94; INDOOR: *r*_*s*_ = −0.015, *p* = 0.887, *n* = 92).

### Release Velocity of the Ball

As expected, due to the larger court in indoor volleyball, mean release velocities of indoor float serves (*x* = 17.79 m/s, SD = 0.99 m/s, *n* = 92) were higher than beach float serves (*x* = 15.14 m/s, SD = 0.85 m/s, *n* = 94).

Both indoor and beach volleyball release velocities differed significantly between individual athletes (Beach: *F*_(5/93)_ = *14.4, p* < *0.001*; Indoor: *F*_(5/91)_ = *26.8, p* < *0.001*). Interestingly, there was a notable correlation between release velocity and expert rating in both studies (beach: *r*_*s*_ = *0.392, p* < 0.001, *n* = 94; indoor: *r*_*s*_ = 0.514, *p* < 0.001, *n* = 92). However, there was no corresponding relationship between release velocity and objective, kinematic-based floating measures. Regression analysis revealed that release velocity did not predict the extent of floating in the horizontal plane. For both dependent variables (mean residuals, anticipation error) and in both studies, the coefficient of determination was very small (all *r*^2^ < 0.033). For the vertical plane, only in the indoor study, a significant effect of release velocity on ball floating was found with more floating related to lower velocities. However, explained variance was quite low (*r*^2^ = 0.126 for mean residuals and *r*^2^ = 0.086 for anticipation error).

## Discussion

The aim of the present study is to analyze the kinematics of ball-flight trajectories of volleyball float serves from elite level players and to develop a measure to quantify float effects. Furthermore, we investigated whether the objectively measured float effect also corresponds to the subjective perception of experts. Therefore, upon performing each serve by every player, the other co-player and the two coaches rated their perceived floating of the ball. Even though there was no complete congruence between the expert raters, there was a high correlation in judging the float effect of float services. Some of the differences between the raters could be due to the slightly different viewing perspectives. Overall, only a few serves were rated as heavily floating, and not a single serve was consistently rated as very heavily floating by all three raters. Also, there was a surprisingly low quota (65%) of valid serves. Presumably, the pressure to produce a heavy float effect and the missing penalization of service errors led the athletes to take a higher risk in serving.

With the anticipation error, we introduce a measure that describes the distance between the actually measured and the anticipated locus of reception. This measure seems appropriate and useful as the random proportion of the ball flight trajectory is expressed as a spatial deviation that is directly linked to the perceptual and motor demands of the reception player. Based on our findings, we conclude that the anticipation error is a useful tool to represent the quality of the float serve, since it is associated with the raters' perception. Values for single serves up to 1.0 m for the horizontal and up to 1.5 m for the vertical plane illustrate the high demands on fast closed loop corrections of the locus of reception resulting from a successful float serve. Due to the longer indoor court and therefore, to the longer ball path length compared to beach volleyball, it is plausible that in the INDOOR study, anticipation errors are somewhat higher because extrapolation of the flight path on the (“anticipated”) locus of reception was required for longer distances, on average.

Whereas the anticipation error quantifies the effect of random movement (floating) on the prediction of the further course of the ball flight path, the mean residual measure quantifies to which extent the ball curve is unpredictable regarding the entire trajectory. Similar to the anticipation error measure, float effects expressed as mean residuals are more pronounced in the flight direction compared to the crosswise direction (horizontal plane).

Although the study was designed to capture float effects close to competition conditions, it should be noted that in the BEACH study, in consultation with the trainer, a net height was used for organizational reasons that differed slightly from the respective competition heights. It can, therefore, not be excluded that the—although small—deviations from the competition heights have an influence on the execution of the float serves and, thus, on the ball flight curves. However, we assume that this influence is only marginal. Compared to the INDOOR study, which was conducted at competition net height, the compromise height chosen in the BEACH study did not lead to a lower number of balls actually floating.

While we could only demonstrate a correlation between the mean residuals and the subjective ratings when looking at the overall floating effect, an analysis broken down by different planes revealed a significant correlation at the horizontal plane for both INDOOR and BEACH and both floating measures, with no corresponding correlations for the vertical plane. We presume, that from the perspectives of the three raters, left/right discrepancies related to the anticipated ball flight trajectory are easier to detect than discrepancies related to the “length” of the ball flight trajectory. The combination of the two plane-specific measures leads to a dilution of the overall correlation between the ratings and the overall measures because the objective floating effect that is added from the vertical plane is not perceived subjectively. The lack of correlation between the expert ratings and the mean residuals in the vertical plane can be explained in two ways. First, quadratic regression could be erroneous because the flight curve could significantly deviate from a parabola due to drag forces. Although the use of a quadratic regression is a simplification, we believe that it is still suitable for detecting the random deviations due to the float effect. In fact, the ball is slowed down by the air resistance during the flight, so the residuals calculated by the quadratic regression are systematically somewhat overestimated. However, we do not see any alternative to our approach. Modeling drag is very difficult and error prone because of the complex relationships between airspeed, ball rotation, and the constantly changing position of the valve in the ball. Second, a float effect in the vertical plane could be harder to detect from a position far behind the baseline—the position from where the experts rated the ball trajectories. Both reasons would lead to lower correlations between objective measures and expert ratings and could have additive effects. Possibly, unexpected deviations can easier be perceived if the observer is positioned on the field. This has implications for the practice of training. If coaches want to reliably detect the float effect in the vertical direction, the position should be carefully chosen. We recommend the center of the side line of the receiving team's field as the best position for observing the ball floating in the vertical direction.

The release velocities measured in the indoor study are, on average, slightly lower, but of the same order of magnitude as the values reported by Huang and Hu (2007; x = 19.7 m/s, SD = 3.7 m/s). The release velocities are also in accordance with the ones Beyer et al. (2013) recommend to enable sudden excursions of the ball's flight (between 15 and 20 m/s). In order to simulate flight path characteristics and thereby float effects, they conducted a wind tunnel study to gather lift and drag data (on two different models of volleyballs) based on indoor volleyball conditions.

Interestingly, there is no evidence in our data that ball release velocity has a strong influence on ball floating; the variance of the float effect explained by ball release velocity is small. In the context of the available literature, this result can be understood to mean that a certain ball velocity is a necessary, but not sufficient, condition for the emergence of erratic ball movement pattern. In fact, all ball release velocities measured in the INDOOR study lie within the range mentioned by Beyer et al. (2013). The velocity-related precondition, therefore, seems to be fulfilled. Quite obviously, how strongly the ball flight curve actually floats, depends to a large extent on the characteristics of further parameters (e.g., ball rotation, ball release angle) and their interaction.

Furthermore, it should be noted that the ball release velocity variation in our study is small. Compared, for example, with the study by Huang and Hu (2007) the standard deviation is less than one third. That is, players show a very high repeatability in the execution of the serve movement with regard to an appropriate ball release velocity. However, if the variable used as predictor (ball release velocity) varies only very little, there can hardly be any covariation in the calculation with the criterion (ball floating).

Similar to our results, standard deviation for landing points of a dimple-type volleyball (Mikasa MVA200) ejected from an impact-type ball ejection device (Asai et al., 2010) with an initial speed of 15 m/s was higher in the flight direction (SD = 1.14 m) than for horizontal dispersion (SD = 0.71 m). However, compared to the float effects (anticipation error) in our study, they found larger effects in both directions. These differences can be explained by the fact that in our measures, the predictable proportion of the ball trajectory is considered by applying polynomial regressions. However, in order to do that, 3D-coordinates of the ball trajectories are required.

What we present here is—to the best of our knowledge for the first time—data that allow a realistic estimate of the magnitude of the float effect in volleyball. With regard to the relevance for competitive volleyball, it should be emphasized that the analyzed ball flight curves originated from very experienced beach and indoor volleyball players.

There is broad consensus in volleyball that the float effect is a performance-relevant phenomenon both in indoor and beach volleyball. The effectiveness of a float serve depends largely on the player hitting the ball in such a way that the release parameters necessary for the float effect are realized. There are practical recommendations on how this can be achieved (Czimek, 2017). However, up to now, there is a lack of evidence as to whether the biomechanical constellations targeted by this technique actually cause the intended float effect and to what extent the respective float effect occurs. With our method, the question of the impact of different ball flight parameters on the ball floating can now be investigated.

For training practice, a measurement system can now be developed that provides a reliable and fast measurement of release angle, speed, and ball rotation, which can be used as augmented feedback. The precise and reliable control of these parameters is obviously difficult even for highly skilled players. One reason for this may be that the internally available information on the execution of the movement is incomplete or “noisy” due to short contact times. This justifies the need for objective feedback.

## Conclusion

The effectiveness of the volleyball float serve lies in the fact that the unpredictability of the ball flight trajectory significantly increases the difficulty of its reception. The greater the deviations from the anticipated locus of reception, the greater the corrective movements required. The suggested regression analysis of the ball trajectories has shown to be an adequate approach to capture the complexity of the float effect and allows to calculate objective measures of ball floating. With the mean residual measure and the anticipation error, two different aspects of ball-flight trajectories of float serves from elite volleyball players were analyzed. We could give evidence—to our knowledge, for the first time in a setting close to competition—that the float effect can cause a significant deviation error, in our study of up to 1.5 m in the vertical and about 1 m in the horizontal direction.

## Data Availability Statement

All relevant data is contained within the article.

## Ethics Statement

The authors declare that all experiments performed in the study do comply with the current laws of Germany.

## Author Contributions

MR, SKü, and KZ contributed in the conception and design of the study. MR, SKi, and KZ acquired the data. SKi organized the database and performed the calculations together with MR. MR and SKü performed the statistical analysis. MR wrote the first draft of the manuscript, KZ and SKü contributed. All authors approved the final version. All authors contributed to the article and approved the submitted version.

## Conflict of Interest

The authors declare that the research was conducted in the absence of any commercial or financial relationships that could be construed as a potential conflict of interest.
